# Phylogenetic Investigation of Norovirus Transmission between Humans and Animals

**DOI:** 10.3390/v12111287

**Published:** 2020-11-10

**Authors:** Nele Villabruna, Ray W. Izquierdo Lara, Judit Szarvas, Marion P. G. Koopmans, Miranda de Graaf

**Affiliations:** 1 Department of Viroscience, Erasmus MC, Wytemaweg 80, 3015CN Rotterdam, The Netherlands; n.villabruna@erasmusmc.nl (N.V.); r.izquierdolara@erasmusmc.nl (R.W.I.L.); m.koopmans@erasmusmc.nl (M.P.G.K.); 2 Research Group for Genomic Epidemiology, Division for Global Surveillance, National Food Institute, Technical University of Denmark, 2800 Kongens Lyngby, Denmark; jusz@food.dtu.dk

**Keywords:** norovirus, phylogeny, animal reservoir

## Abstract

Norovirus infections are a leading cause of acute gastroenteritis worldwide, affecting people of all ages. There are 10 norovirus genogroups (GI-GX) that infect humans and animals in a host-specific manner. New variants and genotypes frequently emerge, and their origin is not well understood. One hypothesis is that new human infections may be seeded from an animal reservoir, as human noroviruses have occasionally been detected in animal species. The majority of these sequences were identified as older GII.4 variants, but a variety of other GIIs and GIs have been detected as well. While these sequences share at least 94% nt similarity with human strains, most of them are >98% identical to human strains. The fact that these strains were detected in animals after they had been detected through human surveillance to be already circulating in humans suggests human-to-animal transmission.

## 1. Introduction

Noroviruses are an important cause of gastroenteritis in humans and animals [1]. Their genome is 7.5 kb in length and organized in three open reading frames (ORF1-3) [2]. ORF1 encodes a polyprotein that is enzymatically cleaved by the viral protease into six proteins, including RNA-dependent RNA polymerase (RdRp). ORF2 and ORF3 encode for the major and minor capsid protein (VP1 and VP2), which make up the virus capsid. VP1 is composed of the conserved shell-domain and the protruding (p)-domain, which contains the receptor binding sites that recognize histo-blood group antigens (HBGAs), and the antigenic sites [3,4]. Based on phylogenetic analysis of VP1 sequences, 10 genogroups have been identified (GI-GX), which are further divided into 49 genotypes, of which some include several variants [5]. Viruses within genogroups I, II, IV, VIII, and IX infect humans, with GI and GII being the most commonly detected genotypes. Viruses from the other genogroups have been found in a broad range of animals including cattle and sheep (GIII), cats and dogs (GIV, GVI, and GVII), rodents (GV), bats (GX), and harbor porpoises (GNA1). Despite this large number of genotypes, viruses within GII.4 are most commonly detected in humans and are responsible for the majority of outbreaks [6,7,8]. Norovirus diversity is additionally increased by recombination events between ORF1 and ORF2, resulting in new strains. New variants, genotypes, and recombinants frequently emerge in the human population, yet their origin is unknown. One hypothesis is that they originate from an animal reservoir. We have previously systematically reviewed serological evidence of transmission between animals and humans and described that more evidence exists for human-to-animal transmission than vice versa [9]. However, given the presence of host-specific noroviruses, the possibility of serological reactivity due to the presence of cross-reactive antibodies cannot be excluded. More conclusive evidence can be gained from virological testing, and although viral RNA of animal strains has not been detected in humans, viral RNA of human GI and GII strains has been detected in fecal material of calves, pigs, birds, captive macaques, dogs, and rodents (reviewed in reference [9]). Most of these animals are also susceptible to human noroviruses under experimental conditions [10]. This implies that animals could be a possible reservoir for human noroviruses. To explore this possibility and investigate the genetic relationship of human noroviruses detected in animals and humans, we have analyzed all human norovirus sequences that, to date, have been found in animal stool samples.

## 2. Materials and Methods

### 2.1. Phylogenetic Analyses

Published sequences of human noroviruses detected in animal feces were collected and searched against the entire GenBank database for DNA sequence (BLASTN). The 20 best hits were downloaded and typed using the Noronet typing tool [11]. Blast hits that were identical to each other were excluded. Sequences from animal inoculation experiments were also excluded. For the phylogeny we used the blast hits as well as sequences of the respective genotypes and variants from the Noronet typing tool reference sequence set (https://www.rivm.nl/mpf/typingtool/norovirus). Alignments were made using MUSCLE [12]. Maximum likelihood trees were created with PhyML v3.0 [13] (http://www.atgc-montpellier.fr/phyml/) and an automated model was selected by Smart Model Selection (SMS [14]) with 100 bootstrap replications. The trees were visualized using FigTree v1.4.3 (http://tree.bio.ed.ac.uk/software/figtree/).

### 2.2. BEAST Analyses

GII.7 and a GII.17 were the only sequences of which the whole VP1 was available and which contained nonsynonymous mutations compared to the most closely related human strains. Therefore, these were used in the BEAST analysis. All complete or near complete GII.7 and GII.17 VP1 sequences were downloaded from GenBank and aligned separately with MUSCLE [12]. The temporal signal of each group of sequences was evaluated with TempEst v1.5.3 [15] and sequence outliers were removed from the final dataset. Bayesian phylogenetic trees based on complete VP1 sequences were inferred using BEAST v1.10.4 [16]. For GII.7 sequences, the final dataset included 29 sequences in the alignment (1560 bp). The general time reversible (GTR) substitution model was used with 4 gamma categories with 3 partition into codon positions to generate an uncorrelated relaxed molecular clock. The tree prior was set as an exponential growth and random sampling. The Markov chain Monte Carlo (MCMC) was set to 50,000,000 generations to ensure convergence. For GII.17 sequences, the final dataset included 764 sequences in the alignment (1484 bp), corresponding to the period between 2013 and 2018 and belonging to the Kawasaki308 cluster. The HKY substitution model and the population size was assumed to be constant throughout its evolutionary history. The MCMC run was set to 400,000,000 generations to ensure convergence. In both datasets, if the day from the collection date was missing, the day was set as the 15th of the given month. If both day and month were missing, the collection date was set as June 15 of the given year. Log files were analyzed in Tracer v1.7.1 to check if ESS values were beyond threshold >200 [17]. The maximum clade credibility tree was constructed with 10% burn-in of the trees using TreeAnnotator v1.10.4. Trees were annotated and visualized using FigTree v1.4.3. The reliability of the branches was supported by 95% highest posterior densities (HPDs).

### 2.3. Mapping of Amino Acid Changes onto 3D Structure

Amino acid changes of GII.7 and GII.17 that were unique to strains found in animals, were mapped onto 3D p-particle structures using EzMol v2.1 [18]. The three-dimensional structure of the GII.7 strain was predicted by homologous modelling using SWISS-MODEL server (available at https://swissmodel.expasy.org/interactive) with default settings. The model was built on the basis of the crystal structure of the p-domain of a GII.17 strain (PDB number 5f4o.1). For GII.7 (KT943504 and KT943505) the predicted 3D p-domain structure was used and for GII.17 (KX356908) the p-domain structure of GII.17 Kawasaki (5LKG). The antigenic epitopes were inferred from those of GII.4 using multiple sequence alignment [17] and information on the HBGA binding site was taken from reference [19].

## 3. Results

### 3.1. Norovirus Strains, Closely Related to Human Noroviruses, Are Found in Animals

Published sequences of human noroviruses detected in animal feces were collected [7], and sample information is summarized in Table 1. Human noroviruses have been found in a variety of mostly asymptomatic animals, of which the domestic pig was the most common species. While three whole genomes have been sequenced (two GII.4 Sydney[P31] from dogs and one GII.17[P17] from a rhesus macaque), most published sequences are short, 200–300 bp in length, and cover the 5′ end of VP1, reflecting commonly used targets for diagnostic RT-PCR assays. Single sequences that cover different parts of ORF1 were not used for phylogenetic analysis but are listed in Table 1.

**Figure 1:**
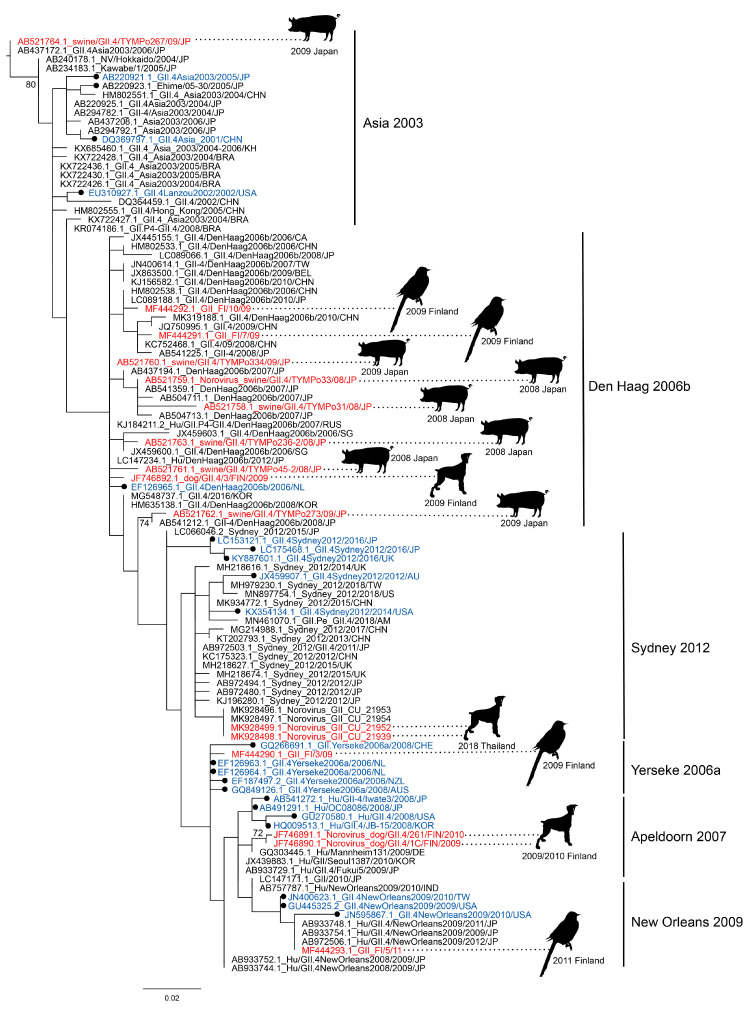
Genetic characterization of GII.4 sequences found in animals. A maximum-likelihood tree based on 223 bp GII.4 sequences was inferred with PhyML v3.0 software using the general time reversible nucleotide substitution model (GTR + G). Sequences that were found in animals (red) were aligned with most closely related human sequences (black) and the reference sequences from the noronet typing tool (blue, black circle). The animal in which norovirus was found as well as the date and country of collection are indicated next to the sequence. The scale bar indicates nucleotide substitutions per site.

Overall, the animal strains are very close or even identical to human strains, ranging from 94% to 100% nt identity. It is worth noting that none of these strains differed enough to be categorized as a new variant. Three sequences belonged to the GI genogroup and all others to GII, GII.4 being the most commonly found genotype. Most GII.4 sequences were typed as older variants, predominantly den Haag 2006b, but also Farmington Hills 2002 (only RdRp), Asia 2003, Yerseke 2006a, Apeldoorn 2007, New Orleans 2009, and more recently GII.4 2012 Sydney (Figure 1). The isolation dates of these samples coincide with the end of the time period that these strains were circulating in the human population (Table 1). Den Haag 2006b was most prevalent in the human population between 2006 and 2008 [21], but the collection dates of the animal samples fell between 2008 and 2009, with the exception of one RdRp sequence, which was collected in 2005. Two studies which included samples of close contact humans with symptoms detected identical GII.4 sequences in dogs and their owners: the two full genome GII.4 Sydney sequences found in Thailand and an unassigned GII.4 in Finland [25,26]. Most studies, unfortunately, did not include samples of close contact humans.

**Figure 2:**
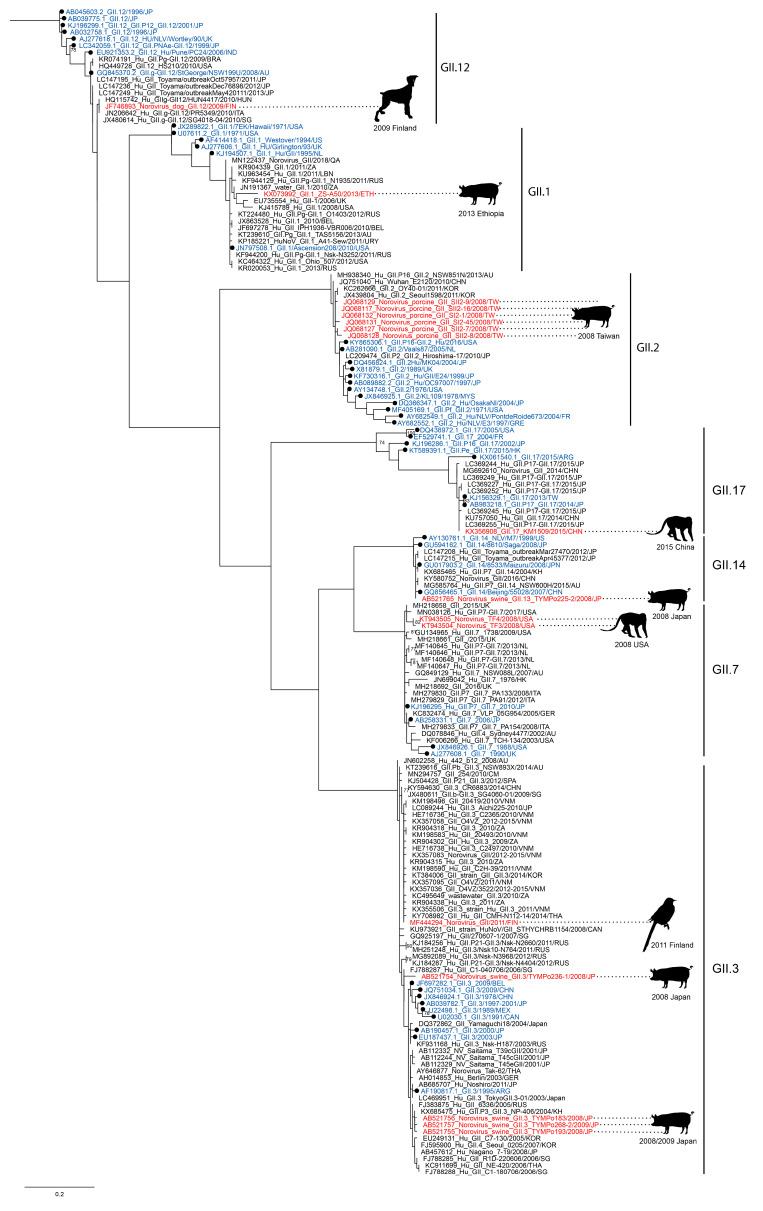
Genetic characterization of non-GII.4 GII sequences found in animals. A maximum-likelihood tree based on 180 bp GII sequences was inferred with PhyML v3.0 software using the general time reversible nucleotide substitution model (GTR + G). Sequences that were found in animals (red) were aligned with most closely related human sequences (black) and the reference sequences from the noronet typing tool (blue, black circle). The animal in which norovirus was found as well as the date and country of collection are indicated next to the sequence. The scale bar indicates nucleotide substitutions per site.

**Figure 3:**
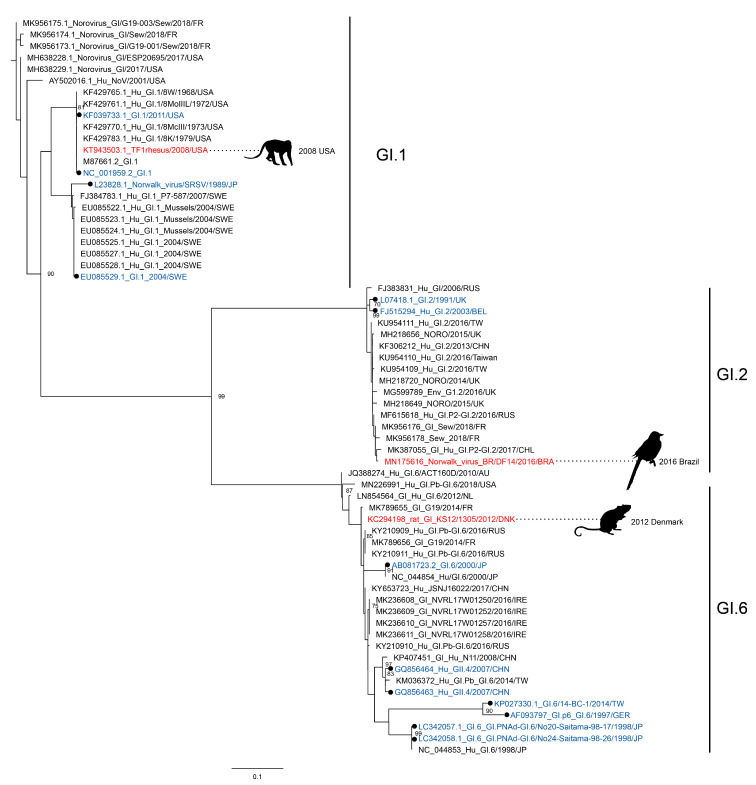
Genetic characterization of GI sequences found in animals. A maximum-likelihood tree based on 271 bp GI sequences was inferred with PhyML v3.0 software using the general time reversible nucleotide substitution model (GTR + G). Sequences that were found in animals (red) were aligned with most closely related human sequences (black) and the reference sequences from the noronet typing tool (blue, black circle). The animal in which norovirus was found as well as the date and country of collection are indicated next to the sequence. The scale bar indicates nucleotide substitutions per site.

While GII.4 was the most commonly found genotype, other GII and GI noroviruses have been detected as well (Figure 2 and Figure 3). Some strains matched the then-circulating strains in the human population, such as GII.3 and GII.17, of which the latter was one of the most prevalent genotype in the period 2014–2015 [34]. Other strains are less frequently found in humans, and their discovery in animals was therefore more surprising. These include the GI genotypes as well as GII.1, GII.2, GII.12, and GII.14 viruses. The recent finding of a GI.1 virus, which was identical to the prototype strain first isolated in 1968, is unexpected. This specific GI.1 is not detected in humans anymore, but newer GI.1 variants are sporadically detected in humans and in sewage [7,35,36]. These findings spark the question of whether the less frequently detected GII and GI viruses continue to circulate undetected in humans and animals.

### 3.2. Molecular Clock Phylogeny of GII.7 and GII.17 Genotypes

**Figure 4:**
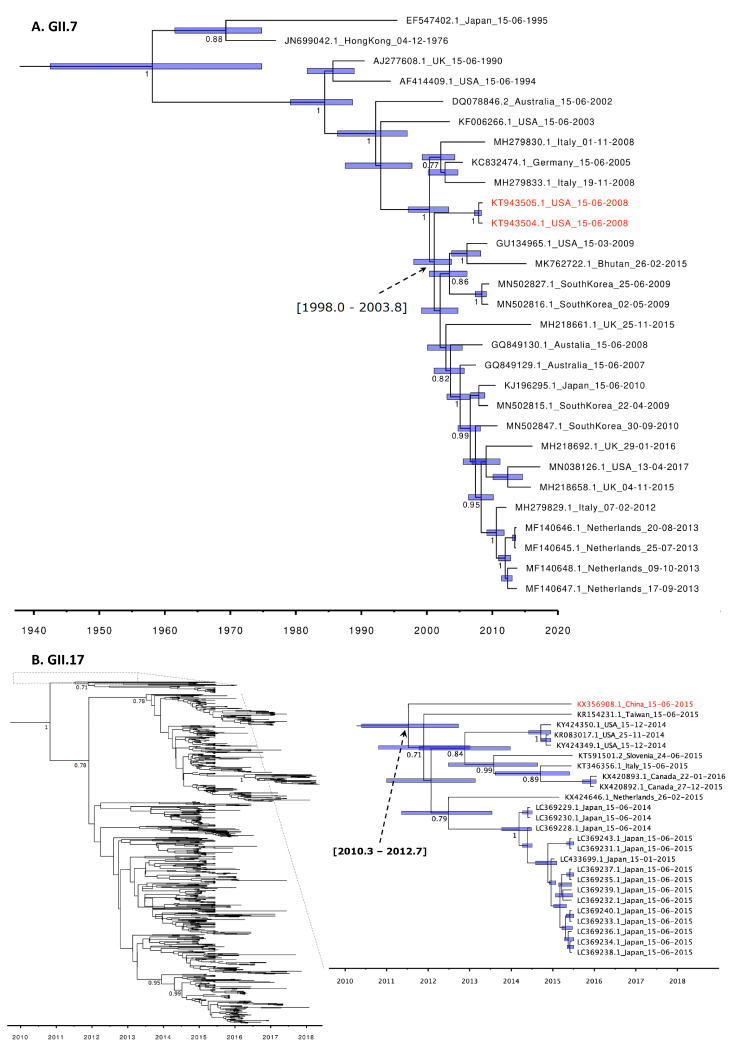
Molecular clock phylogeny of the complete VP1 gene sequences for GII.7 (**A**) and GII.17 (Kawasaki308 cluster) (**B**) constructed by the Bayesian Markov chain Monte Carlo (MCMC) method. Sequences in red indicate sequences from animal origin. Node bars indicate the 95% HPDs of the time of the most common recent ancestor. Numbers in the nodes show the posterior densities (only values > 0.7 are shown). The expanded GII.17 tree is shown in Supplementary Figure S1.

To investigate the evolutionary relationship between noroviruses detected in humans and animals and to estimate how long ago they diverged, we conducted a BEAST analysis. Of the noroviruses found in animals, the complete VP1 sequences were only available for viruses belonging to GII.4 (MK928498-99), GI.1 (KT943503), GI.6 (KC294198), GII.17 (KX356908), and GII.7 (KT943504/5). Of these, two GII.7 sequences and a GII.17 sequence (all found in rhesus macaques) were the only sequences with nonsynonymous mutations compared to the most closely related human strains. To determine the time to the most recent common ancestor (tMRCA) of the rhesus macaque-derived VP1 gene sequences to those found in humans, we performed separate BEAST analysis for these two genotypes. The estimated time to the MRCA of the rhesus macaque GII.7 to known human strains was shown to be around the end of the year 2000 (between 1998.0 and 2003.8, 95% HPD), eight years before the rhesus macaque GII.7 was detected (Figure 4A). For the rhesus macaque GII.17 this was around 2011 (between 2010.3 and 2012.7, 95% HPD), four years before the rhesus macaque GII.17 strain was detected, and the tMRCA predated the GII.17[P17] outbreaks in humans during the winter of 2014–2015 (Figure 4B, Supplementary Figure S1). However, it should be noted that the tMRCA 95% HPD interval is large and does not necessarily predate the tMRCA solely human GII.17 strains within the same clade.

### 3.3. Animal GII.7 and GII.17 Sequences Contain Amino Acid Changes That Are Located either in or Adjacent to Antigenic Epitopes

**Figure 5:**
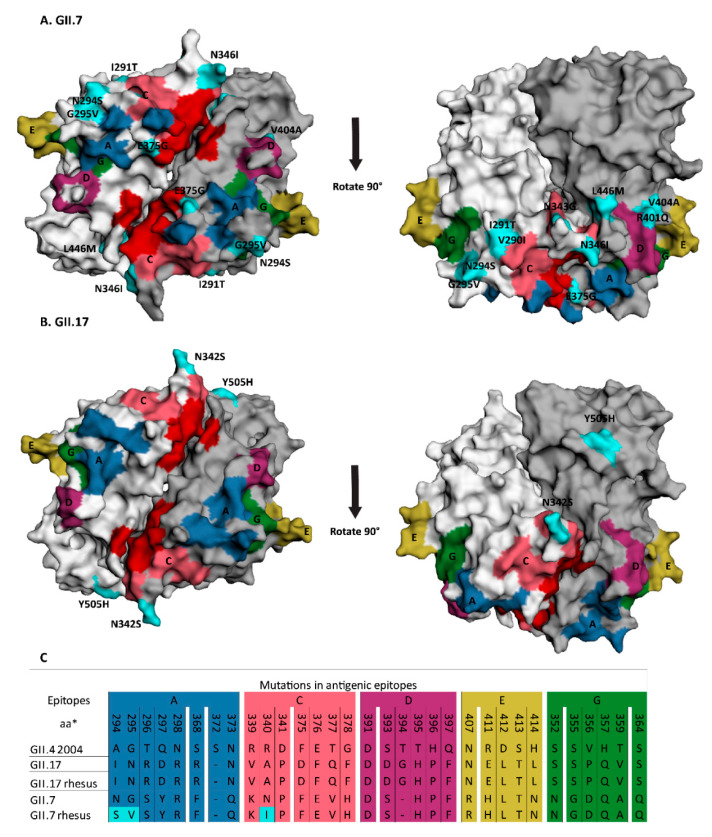
Mapping of amino acid differences between human strains found in animals versus humans onto p-dimers (the two subunits are shown in two shades of grey, top-view). Amino acid changes exclusively found in the strains detected in animals are colored in turquoise. Predicted HBGA binding sites are marked in red, and the epitopes A-G in color. Changes were mapped onto the p-domain GII.7 3D structure predicted by SWISS-MODEL on the basis of the crystal structure of the p-domain of a GII.17 strain (PDB number 5f4o.1) for GII.7 (KT943504 and KT943505) (**A**) and GII.17 Kawasaki (5LKG) for GII.17 (KX356908) (**B**). The alignment was performed with MEGA ClustalW and shows the amino acids defining the antigenic sites [17] that differ between strains found in animals and humans (**C**). Amino acid numbering was based on GII.4 2004. Putative binding sites were taken from reference [37].

Amino acid changes in the exposed protruding p-domain of the capsid can lead to differences in either HBGA binding or antigenic drift [24]. To identify whether the 13 and 4 amino acid changes found in GII.7 and GII.17 VP1 sequences from macaques are close to an antigenic epitope or receptor binding site, we mapped their location onto the predicted 3D GII.7 structure of the p-domain and a 3D GII.17 p-domain structure, respectively. The antigenic epitopes were predicted based on an alignment with GII. 4 sequences. The GII.7 sequence had several amino acid changes that were located either within a predicted antigenic epitope or in close proximity (Figure 5A). Three changes were located directly in the predicted antigenic epitopes (Figure 5C, Supplementary Figure S2): N294S and G295V in epitope A, and N346I in epitope C. Another seven were in close proximity to predicted epitopes: E375G was situated right next to the HBGA binding site, N343G, V290I and I291T were adjacent to epitope C, and V404A, R401Q, and L446M were next to epitope D. Two changes, I478V and Y514H, were on the surface but distant from any epitopes, while T54N was located outside of the p-domain. Of the four changes found in the GII.17 sequence, N342S was the only one in proximity to epitope C (Figure 5B,C, Supplementary Figure S2). Y505H was on the surface but distant from any predicted epitopes. P280S and G282D are not surface exposed. Thus, some of the observed mutations potentially affect HBGA-binding specificity and antigenicity.

## 4. Discussion

Norovirus genome sequences that are very similar or even identical to those of human strains have been detected in animals all over the world. The timing of detection of human-like sequences in animals almost invariably coincides with the circulation of the matching variants in the human population, and most sequences were highly similar, indicating a recent spillover. This was especially visible for GII.4 viruses, which—in the human population—undergo epochal evolution leading to emergence of antigenically distinct variants every few years, replacing the previous viruses [25]. For the GII.4 viral genomes detected in animals, assuming that the direction of transmission was from humans to animals seems most plausible, as the GII.4 variants were circulating in humans before they were found in animals. This was also the case for two studies that analyzed human and animal virus sequences from the same household [18,19].

The epidemiology of non-GII.4 genotype noroviruses is distinct. Non-GII.4 viruses also have a global distribution, and cause sporadic infections and outbreaks [23], but do not evolve as rapidly as GII.4 variants and do not show the pattern of variant replacement [24]. Nevertheless, our analysis showed that most GII sequences found in animals were also very close or identical to human strains, arguing against long-term circulation in animals. It should, however, be noted that sequence information was often limited to very short fragments that are commonly used as diagnostic targets, as the sequences cover conserved regions. It is intriguing that the two longer sequences belonging to GII.7 and GII.17 that were available were the viruses with the most diverged nucleotide sequence compared to human variants. They were both found in captive macaques, but no information about humans or contaminated food from those centers was available. The BEAST analysis placed the most recent common ancestor to human isolates four and eight years before their detection in macaques, revealing a considerable temporal and genetic gap of these genotypes. For the GII.17[P17] strain detected in macaques the tMRCA predated their detection in humans. This can be explained either by lack of knowledge about the GII.7 and GII.17 diversity in humans or by the undetected circulation of these genotypes in a non-human reservoir. GII.7, and to a lesser degree GII.17, had accumulated amino acid changes that were located in regions predicted to define antigenicity of norovirus, thereby possibly resulting in an adapted phenotype. The epitopes in GII.7 and GII.17 were inferred from those of GII.4. It should be noted that these have only been established as antigenic epitopes in GII.4 and not for any other genotype. However, comparison of capsid sequences indicates that GII.17 is evolving at previously defined GII.4 antibody epitopes [37]. In our analysis, the rhesus macaque GII.17 strain only had one mutation near the HBGA binding site compared to the most closely related strains detected in humans. Saliva binding studies using recombinant protein showed that the rhesus macaque GII.17 strain binds to human saliva samples with significantly lower binding signals than a similar human GII.17 strain with two mutations near the HBGA binding site [26]. Thus, animals can harbor human norovirus strains that potentially have antigenic and binding properties that differ from those detected in humans.

As the interface between wildlife and domesticated animals and humans is expanding, the risk of pathogens jumping the species barrier increases. While much of current virus research is focused toward transmission from animals-to-humans, our results show that the reverse should not be neglected, as it might have consequences for pathogen dynamics in humans as well as in animals. How often human-to-animal transmission of norovirus occurs, and if they are single events or if human strains circulate continuously in some animal reservoir, needs to be further addressed. Given the prevalence of host-specific viruses in several of the species of animals in which human norovirus sequences were detected, there is at least in theory the potential for recombination in case of dual infections. The question of whether human noroviruses in animals or recombinant human animal norovirus genomes are transmitted back into the human population, and therefore have an impact on (re)-emergence of noroviruses, remains to be answered.

## Figures and Tables

**Table 1:** Information about human norovirus sequences found in animals.

VP1 = Virus Capsid Protein 1 (ORF2), VP2 = Virus Capsid Protein 2 (ORF3), RdRp = RNA-Dependent RNA Polymerase									
Accession Number	Host	Location	Year	Circulation Based on References [20,21]	Norovirus Typing	Sequence Length (bp)	Genome Region Covered	Similarity to Best Blast Hit	Ref
MF444290	Bird	Finland	2009	NA	GII.4 could not assign	223	VP1	222/223 (99.5%)	[22]
MF444291	Bird	Finland	2009	NA	GII.4 could not assign	223	VP1	222/223 (99.5%)	[22]
MF444292	Bird	Finland	2009	2006–8	GII.4 Den Haag 2006b	223	VP1	222/223 (99.5%)	[22]
MF444293	Bird	Finland	2011	2009–12	GII.4 New Orleans 2009	223	VP1	223/223 (100%)	[22]
JQ068133 *	Pig	Taiwan	2008	2006–8	GII.4 Den Haag 2006b	239	RdRp/VP1	239/239 (100%)	[23]
AB521758-63	Pig	Japan	2008/9	2006–8	GII.4 Den Haag 2006b	302	VP1	298/302 (98.9%)–302/302 (100%)	[24]
AB521764	Pig	Japan	2009	2002–6	GII.4 Asia 2003	302	VP1	302/302 (100%)	[24]
JF746890^1^	Dog	Finland	2009	NA	GII.4 could not assign	283	VP1	281/283 (99.3%)	[25]
JF746891	Dog	Finland	2010	NA	GII.4 could not assign	283	VP1	281/283 (99.3%)	[25]
JF746892	Dog	Finland	2009	2006–8	GII.4 Den Haag 2006b	283	VP1	281/283 (99.3%)	[25]
MK928498-99 ^1^	Dog	Thailand	2018	2012–20	GII.4 Sydney 2012[P31]	7513	Genome	7457/7513 (99.3%)	[26]
EF175441 **	Pig	Canada	2005	2002–4	GII.P4 Farmington Hills 2002	172	RdRp	172/172 (100%)	[27]
CE-M-05–0114 **^,6^	Pig	Canada	2005	NA	GII could not assign	172	RdRp	172/172 (100%)	[27]
CE-M-06–0013 **^,6^	Pig	Canada	2005	2002–4	GII.P4 Farmington Hills 2002	172	RdRp	169/172 (98.3%)	[27]
CE-M-06–0509 **^,6^	Cattle	Canada	2005	2006–8	GII.P4 Den Haag 2006b	172	RdRp	168/172 (98.3%)	[27]
GU556160-66 **	Pig	Taiwan	2008	2006–08	GII.P4 Den Haag 2006b	260	RdRp	241/256 (94%)	[23]
MN175616	Bird	Brazil	2016	NA	GI.2	492	VP1	475/478 (99.4%)	[28]
KT943503	Macaque ^2^	USA	2008	NA	GI.1	1670	VP1-VP2	1670/1670 (100%)	[29]
KC294198	Rat ^3^	Denmark	2012	NA	GI.6[GI.Pb]	4012	RdRp-VP1/2	3959/3993 (99.1%)	[30]
MF444294	Crow ^4^	Finland	2011	NA	GII.3	223	VP1	223/223 (100%)	[22]
KX073992	Pig	Ethiopia	2013	NA	GII.1	291	VP1	279/291 (95.9%)	[31]
KT943504	Macaque	USA	2008	NA	GII.7	1623	VP1	1570/1632 (96%)	[29]
KT943505	Macaque ^2^	USA	2008	NA	GII.7	1623	VP1	1570/1632 (96%)	[29]
JF746893	Dog	Finland	2009	NA	GII.12	283	VP1	282/283 (99.7%)	[25]
KX356908	Macaque	China	2015	2014–15	GII.17[P17]	7556	Genome	7496/7556 (99.2%)	[32]
AB521765	Pig	Japan	2008	NA	GII.14	302	VP1	291/302 (96.4%)	[24]
AB521754-57	Pig	Japan	2008	NA	GII.3	302	VP1	297/302 (98.3%)–301/302 (99.7%)	[24]
JQ068117-132	Pig	Taiwan	2008	NA	GII.2	239	VP1	238/239 (99.6%)	[23]
HM035148 **	Macaque ^2^	USA	2008	NA	GII.7 could not assign ^5^	274	RdRp	259/274 (94.5%) to GII.P7	[29,33]
MN175617 **	Bird	Brazil	2016	NA	GII.P31 could not assign ^5^	438	p48	416/419 (99.3%) (gap)	[28]

^1^ Had identical nt identity with sequences found in associated human; ^2^ *Rhesus macaque*; ^3^ *Rattus norvegicus*; ^4^ *Corvus corone cornix*, for other birds no more details were given; ^5^ Outside typing region. Genotype based on closest blast hit; ^6^ Sequence was only published in paper but without accession number; * Was not included in phylogeny because coverage of VP1 was too short for alignment; ** Were not included because they covered regions outside ORF2.

## Supplementary Materials

The following are available online at https://www.mdpi.com/1999-4915/12/11/1287/s1, Figure S1: Extended tree of molecular clock phylogeny of the complete VP1 gene sequences for GII.7, Figure S2: Multiple amino acid sequence alignment of the norovirus GII.7 and GII.17 p-domain.

## References

[1] Vinje J. (2015). Advances in laboratory methods for detection and typing of norovirus. J. Clin. Microbiol..

[2] Thorne L.G., Goodfellow I.G. (2014). Norovirus gene expression and replication. J. Gen. Virol..

[3] Tan M., Jiang X. (2005). Norovirus and its histo-blood group antigen receptors: An answer to a historical puzzle. Trends Microbiol..

[4] Bok K., Abente E.J., Realpe-Quintero M., Mitra T., Sosnovtsev S.V., Kapikian A.Z., Green K.Y. (2009). Evolutionary dynamics of gii.4 noroviruses over a 34-year period. J. Virol..

[5] Chhabra P., de Graaf M., Parra G.I., Chan M.C.-W., Green K., Martella V., Wang Q., White P.A., Katayama K., Vennema H. (2019). Updated classification of norovirus genogroups and genotypes. J. Gen. Virol..

[6] Tran T.N.H., Trainor E., Nakagomi T., Cunliffe N.A., Nakagomi O. (2013). Molecular epidemiology of noroviruses associated with acute sporadic gastroenteritis in children: Global distribution of genogroups, genotypes and gii.4 variants. J. Clin. Virol..

[7] van Beek J., de Graaf M., Al-Hello H., Allen D.J., Ambert-Balay K., Botteldoorn N., Brytting M., Buesa J., Cabrerizo M., Chan M. (2018). Molecular surveillance of norovirus, 2005–2016: An epidemiological analysis of data collected from the noronet network. Lancet Infect. Dis..

[8] Cannon J.L., Barclay L., Collins N.R., Wikswo M.E., Castro C.J., Magaña L.C., Gregoricus N., Marine R.L., Chhabra P., Vinjé J. (2017). Genetic and epidemiologic trends of norovirus outbreaks in the united states from 2013 to 2016 demonstrated emergence of novel gii. 4 recombinant viruses. J. Clin. Microbiol..

[9] Villabruna N., Koopmans M., De Graaf M. (2019). Animals as reservoir for human norovirus. Viruses.

[10] Todd K.V., Tripp R.A. (2019). Human norovirus: Experimental models of infection. Viruses.

[11] Kroneman A., Vega E., Vennema H., Vinje J., White P.A., Hansman G., Green K., Martella V., Katayama K., Koopmans M. (2013). Proposal for a unified norovirus nomenclature and genotyping. Arch. Virol..

[12] Edgar R.C. (2004). Muscle: Multiple sequence alignment with high accuracy and high throughput. Nucleic Acids Res..

[13] Guindon S., Dufayard J.F., Lefort V., Anisimova M., Hordijk W., Gascuel O. (2010). New algorithms and methods to estimate maximum-likelihood phylogenies: Assessing the performance of phyml 3.0. Syst. Biol..

[14] Lefort V., Longueville J.E., Gascuel O. (2017). Sms: Smart model selection in phyml. Mol. Biol. Evol..

[15] Rambaut A., Lam T.T., Max Carvalho L., Pybus O.G. (2016). Exploring the temporal structure of heterochronous sequences using tempest (formerly path-o-gen). Virus. Evol..

[16] Suchard M.A., Lemey P., Baele G., Ayres D.L., Drummond A.J., Rambaut A. (2018). Bayesian phylogenetic and phylodynamic data integration using beast 1.10. Virus. Evol..

[17] Rambaut A., Drummond A.J., Xie D., Baele G., Suchard M.A. (2018). Posterior summarization in bayesian phylogenetics using tracer 1.7. Syst. Biol..

[18] Reynolds C.R., Islam S.A., Sternberg M.J.E. (2018). Ezmol: A web server wizard for the rapid visualization and image production of protein and nucleic acid structures. J. Mol. Biol..

[19] Tan M., Jiang X. (2010). Norovirus gastroenteritis, carbohydrate receptors, and animal models. PLoS Pathog..

[20] Siebenga J.J., Vennema H., Zheng D.P., Vinjé J., Lee B.E., Pang X.L., Ho E.C.M., Lim W., Choudekar A., Broor S. (2009). Norovirus illness is a global problem: Emergence and spread of norovirus gii. 4 variants, 2001–2007. J. Infect. Dis..

[21] Tohma K., Lepore C.J., Gao Y., Ford-Siltz L.A., Parra G.I. (2019). Population genomics of gii.4 noroviruses reveal complex diversification and new antigenic sites involved in the emergence of pandemic strains. MBio.

[22] Summa M., Henttonen H., Maunula L. (2018). Human noroviruses in the faeces of wild birds and rodents-new potential transmission routes. Zoonoses Public Health.

[23] Chao D.Y., Wei J.Y., Chang W.F., Wang J., Wang L.C. (2012). Detection of multiple genotypes of calicivirus infection in asymptomatic swine in taiwan. Zoonoses Public Health.

[24] Nakamura K., Saga Y., Iwai M., Obara M., Horimoto E., Hasegawa S., Kurata T., Okumura H., Nagoshi M., Takizawa T. (2010). Frequent detection of noroviruses and sapoviruses in swine and high genetic diversity of porcine sapovirus in japan during fiscal year 2008. J. Clin. Microbiol..

[25] Summa M., von Bonsdorff C.H., Maunula L. (2012). Pet dogs-a transmission route for human noroviruses?. J. Clin. Virol..

[26] Charoenkul K., Nasamran C., Janetanakit T., Tangwangvivat R., Bunpapong N., Boonyapisitsopa S., Suwannakarn K., Theamboonler A., Chuchaona W., Poovorawan Y. (2020). Human norovirus infection in dogs, thailand. Emerg. Infect. Dis..

[27] Mattison K., Shukla A., Cook A., Pollari F., Friendship R., Kelton D., Bidawid S., Farber J.M. (2007). Human noroviruses in swine and cattle. Emerg. Infect. Dis..

[28] Duarte M.A., Silva F., João M., Brito C.R., Teixeira D.S., Melo F.L., Ribeiro B.M., Nagata T., Campos F.S. (2019). Faecal virome analysis of wild animals from brazil. Viruses.

[29] Farkas T. (2016). Natural norovirus infections in rhesus macaques. Emerg. Infect. Dis..

[30] Wolf S., Reetz J., Johne R., Heiberg A.C., Petri S., Kanig H., Ulrich R.G. (2013). The simultaneous occurrence of human norovirus and hepatitis e virus in a norway rat (rattus norvegicus). Arch. Virol..

[31] Sisay Z., Djikeng A., Berhe N., Belay G., Abegaz W.E., Wang Q.H., Saif L.J. (2016). First detection and molecular characterization of sapoviruses and noroviruses with zoonotic potential in swine in ethiopia. Arch. Virol..

[32] Liu B., Tao Y., Li C., Li X., Liu J., He Z., Xia M., Jiang X., Tan M., Liu H. (2016). Complete genome sequence of a gii.17 norovirus isolated from a rhesus monkey in china. Genome Announc..

[33] Farkas T., Cross R.W., Hargitt I.E., Lerche N.W., Morrow A.L., Sestak K. (2010). Genetic diversity and histo-blood group antigen interactions of rhesus enteric caliciviruses. J. Virol..

[34] de Graaf M., van Beek J., Vennema H., Podkolzin A.T., Hewitt J., Bucardo F., Templeton K., Nordgren J., Reuter G., Lynch M. (2015). Emergence of a novel gii.17 norovirus—End of the gii.4 era?. Euro. Surveill..

[35] Zhou N., Lin X., Wang S., Tao Z., Xiong P., Wang H., Liu Y., Song Y., Xu A. (2016). Molecular epidemiology of gi and gii noroviruses in sewage: 1-year surveillance in eastern china. J. Appl. Microbiol..

[36] Mans J., Netshikweta R., Magwalivha M., Van Zyl W.B., Taylor M.B. (2013). Diverse norovirus genotypes identified in sewage-polluted river water in south africa. Epidemiol. Infect..

[37] Lindesmith L.C., Kocher J.F., Donaldson E.F., Debbink K., Mallory M.L., Swann E.W., Brewer-Jensen P.D., Baric R.S. (2017). Emergence of novel human norovirus gii.17 strains correlates with changes in blockade antibody epitopes. J. Infect. Dis..

