# Supplementary material for: Phylogenetic Investigation of Norovirus Transmission between Humans and Animals
Source: Viruses. 2020 Nov 10;12(11):1287. doi: 10.3390/v12111287 (PMC7698157; doi:10.3390/v12111287)
Supplement: Supplementary file 1 [file viruses-12-01287-s001.zip › supplementary materials/Figure Legends.docx]

Supplementary material

Figure Legends

Supplementary Figure 1

The extended molecular clock phylogeny of the complete VP1 gene sequences for GII.17 (Kawasaki308 cluster) constructed by the Bayesian MCMC method. Number in the nodes show the posterior probabilities (only values >0.7 are shown). The scale bar indicates nucleotide substitutions per site.

Supplementary Figure 2

Multiple amino acid sequence alignment of the norovirus GII.7 and GII.17 p-domain. Positions of GII.4 antigenic epitopes A – G are coloured in the same code as in Figure 5.
