# Supplementary figures and images for: Phylogenetic Investigation of Norovirus Transmission between Humans and Animals
Source: Viruses. 2020 Nov 10;12(11):1287. doi: 10.3390/v12111287 (PMC7698157; doi:10.3390/v12111287)

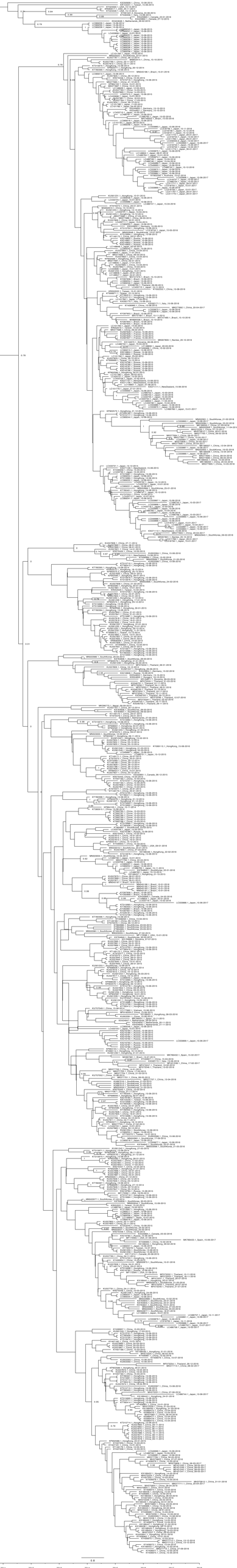

Supplement: Supplementary file 1 [file viruses-12-01287-s001.zip › supplementary materials/Figure_S1.pdf]
