# Supplementary material for: Phylogenetic Investigation of Norovirus Transmission between Humans and Animals
Source: Viruses. 2020 Nov 10;12(11):1287. doi: 10.3390/v12111287 (PMC7698157; doi:10.3390/v12111287)
Supplement: Supplementary file 1 [file Figure_S2.pdf › supplementary materials/Figure_S2.pdf]

```
                                 280       290       300       310       320       330       340       350
                         .....|....|....|....|....|....|....|....|....|....|....|....|....|....|....|...
GII.4_Sydney2012         GTTQLSPVNICTFRGDVTHITG---SRNYTMNLASQNWNNYDPTEEIPAPLGTPDFVGKIQGVLTQTTR---TDGSTRGH
GII.4_2004_HQ456320      GTTQLSPVNICTFRGDVTHIAG---TQNYTMNLASQNWNNYDPTEEIPAPLGTPDFVGKIQGVLTQTTR---RDGSTRGH
GII.17_KU561253          GTTQLLPSGICAFRGRVTAQIN--QRDRWHMQLQNLNGTTYDPTDDVPAPLGTPDFKGVVFGMVSQRNVGNDAPGSTRAQ
GII.17_Rhesus_KX356908   GTTQLLSSDICAFRGRVTAQIN--QRDRWHMQLQNLNGTTYDPTDDVPAPLGTPDFKGVVFGMVSQRNVGSDAPGSTRAQ
GII.7_MF140648           GTTPLLAVNICKFKGEVIAKNGDVRSYRMDMEITNTDGTPIDPTEDTPGPIGSPDFQGILFGVASQRNKNEQNP-ATRAH
GII.7_Rhesus_KT943504/5  GTTPLLAVNICKFKGEITAKSVDVRSYRMDMEITNTDGTPIDPTEDTPGPIGSPDFRGILFGVASQRNKGEQIP-ATRAH

                             360       370       380       390       400       410       420
                         ....|....|....|....|....|....|....|....|....|....|....|....|....|.
GII.4_Sydney2012         KATVYTGSADFAPKLGRVQFETDTDHDFEANQNTKFTPVGVIQDGSTTHRNEPQQWVLPSYSGRNTP
GII.4_2004_HQ456320      KATVSTGSVHFTPKLGSVQFSTDTSNDFETGQNTRFTPVGVVQDGSTTHQNEPQQWVLPNYSGRDSH
GII.17_KU561253          QAWVSTYSPQFVPKLGSVNLRISD-NDDFQFQPTKFTPVGVNDDDDGHTFR---QWELPNYSGELTL
GII.17_Rhesus_KX356908   QAWVSTYSPQFVPKLGSVNLRISD-NDDFQFQPTKFTPVGVNDDDDGHPFR---QWELPNYSGELTL
GII.7_MF140648           EADINTGGDQYAPKLAQVKFFSES-QDFEVHQPTVFTPVGVAGDSS-HPFR---QWVLPRYGGHLTN
GII.7_Rhesus_KT943504/5  EANINTGGDQYAPKLAQVKFFSGS-QDFEVHQPTVFTPVGVAGDSS-HPFQ---QWALPRYGGHLTN
```
